# Localized Colitis Associated With Cholecystectomy in Multiple Sclerosis Patient Treated With Ocrelizumab

**DOI:** 10.7759/cureus.61353

**Published:** 2024-05-30

**Authors:** Rhea Chowdhury, Vladimir Neychev

**Affiliations:** 1 Surgery, University of Central Florida College of Medicine, Orlando, USA

**Keywords:** immunotherapy adverse effect, preoperative considerations, postoperative inflammation, ocrelizumab, localized colitis, patients with multiple sclerosis

## Abstract

Our understanding of multiple sclerosis (MS) has led to the development of new therapeutic strategies, including ocrelizumab, a third-generation humanized anti-CD20 antibody. Ocrelizumab is largely well tolerated with favorable effectiveness, however, it has been associated with reports of colitis presenting weeks to months following infusion. We present a case of severe localized colitis in the setting of recent surgery and chronic ocrelizumab use. High-dose IV hydrocortisone was initiated, and the patient showed gradual improvement. Repeat imaging after discharge showed near-complete resolution of the patient’s condition. This case aims to increase awareness of possible postoperative severe localized colitis in MS patients on Ocrevus.

## Introduction

Treatment of multiple sclerosis (MS) has increasingly relied on B-cell-depleting monoclonal antibodies [1]. Ocrelizumab (Ocrevus^®^) is a humanized anti-CD20 monoclonal antibody approved by the FDA in 2017 for patients with relapsing-remitting and primary progressive forms of MS. It has been found to slow disease progression, as defined by clinical and imaging findings, of primary progressive MS. Compared to other anti-CD20 drugs, such as rituximab, the first with efficacy against MS, ocrelizumab is expected to be less immunogenic with repeat infusions, potentially conferring a preferable benefit-risk profile [1,2]. Currently, Ocrevus serves as a first-line treatment for patients with highly active MS or those refractory to other treatments [3]. The most reported adverse effects of ocrelizumab use, from the OPERA I and II and ORATORIO trials when compared to placebo groups, included a higher incidence of upper respiratory infections, a higher proportion of herpesvirus infections, a higher prevalence of infusion-related reactions, and a possible increased rate of malignancy [3,4]. In addition, there has been an increasing body of evidence in the form of case series demonstrating noninfectious non-ischemic colitis being an off-label side effect of Ocrevus; however, in these cases the colitis was found to be pronounced, extending from the transverse colon to the splenic flexure, down to the sigmoid colon [5,6].

## Case presentation

**Figure 1:**
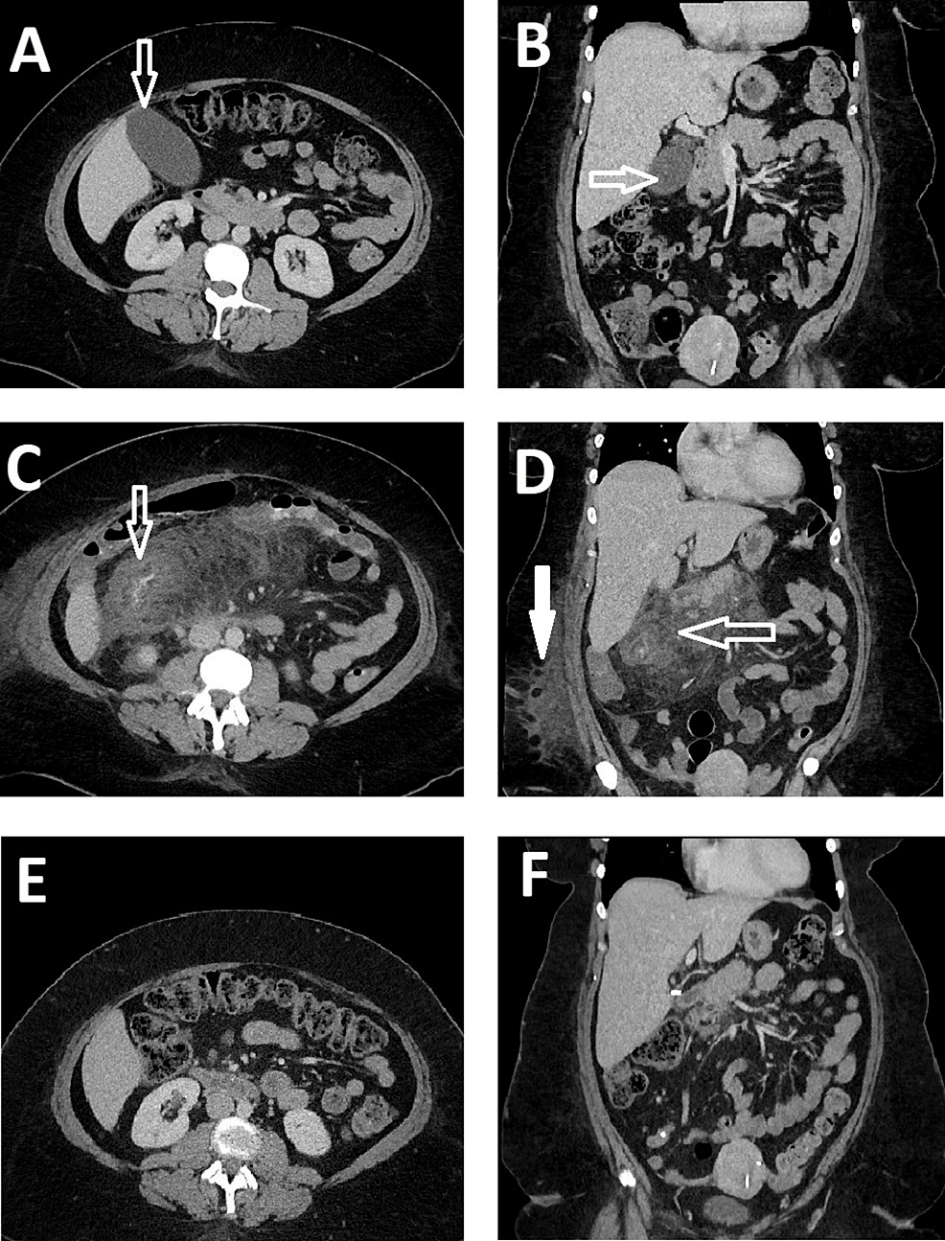
Preoperative and postoperative CT abdomen and pelvis demonstrating postoperative localized colitis A) Representative axial image of the preoperative CT scan of the abdomen and pelvis with a hollow arrow pointing at a distended gallbladder; B) Representative coronal image of the preoperative CT scan of the abdomen and pelvis with a hollow arrow pointing at a distended gallbladder; C) Representative axial image of the CT scan of the abdomen and pelvis on postoperative day four with a hollow arrow pointing at severe inflammatory changes of the hepatic flexure at the proximal third of the transverse colon with stranding of the dependent visceral and retroperitoneal fatty tissue; D) Representative coronal image of the CT scan of the abdomen and pelvis on postoperative day four with a hollow arrow pointing at severe inflammatory changes of the hepatic flexure at the proximal third of the transverse colon with stranding of the dependent visceral and retroperitoneal fatty tissue. The solid arrow is pointing at the stranding of the peripheral fatty tissue; E) Representative axial image, and F) Representative coronal image of the CT scan of the abdomen and pelvis one month postoperatively with complete resolution of the severe localized inflammatory changes of the hepatic flexure at the proximal third of the transverse colon and dependent fatty tissue.

A 47-year-old woman presented to the emergency department with a clinical and paraclinical picture of acute calculous cholecystitis (Figures 1A-1B). The patient had a medical history of gastroesophageal reflux disease (GERD) treated by omeprazole 20 mg, migraines treated by galcanezumab 120 mg every 30 days, hypertension (HTN) treated by lisinopril 10 mg and metoprolol succinate 25 mg, mild pulmonary hypertension, well-controlled MS on Ocrevus IV infusion every six months for the last 18 months, and polycystic ovary syndrome (PCOS) treated by metformin 500 mg. She had no past surgical history.

The surgical intervention consisted of laparoscopic cholecystectomy with indocyanine green fluorescent intraoperative cholangiography (ICG). The gallbladder was sent to pathology, and the results were consistent with cholecystitis secondary to cholelithiasis. She tolerated the procedure well and was discharged on postoperative day one with stable vital signs, appropriate postoperative labs, passing flatus, and tolerating a solid diet.

She returned to the ED four days postoperatively for symptoms of worsening epigastric pain, low-grade fever, and nausea with one episode of vomiting. CT abdomen and pelvis revealed severe inflammatory changes of the hepatic flexure at the proximal third of the transverse colon with significant colonic wall edema, without evidence of perforation. There was prominent edema and stranding of the dependent visceral and retroperitoneal fatty tissue, gastric antrum, duodenum, and pancreatic head. The liver, cholecystectomy bed, and biliary tree appeared largely intact and uninvolved. She was admitted for management and resuscitation. Blood cultures were drawn, and empiric antibiotics started. During her hospital admission, a thorough workup was done to assess for the possible etiology of the inflammatory changes, including GI, viral, and bacterial panels, which were all negative. CT abdomen and pelvis with IV and oral contrast confirmed the severe inflammatory changes of the proximal duodenum and hepatic flexure/transverse colon without perforation (Figures 1C-1D). The neurology team was consulted, and possible surgery-associated colitis of the localized ascending and transverse colon was established as the working diagnosis of exclusion. Treatment with high-dose IV corticosteroids was started. The follow-up labs and serial abdominal exams showed a decrease of WBCs from 15.0x10^3^/uL on admission to 11.64x10^3^/uL, and resolution of her abdominal pain. The patient was discharged on postoperative day 13.

At her outpatient follow-up visit, she was symptom-free with unremarkable labs, and a CT scan of the abdomen and pelvis showed complete resolution of the localized right upper quadrant colonic and abdominal soft tissue inflammation (Figures 1E-1F). After consultation with her neurologist, a decision was made to continue ocrelizumab management of her MS with close monitoring.

## Discussion

Advances in our understanding of MS pathophysiology resulted in the development of novel management strategies targeting CD20 on B lymphocytes. Ocrelizumab is an anti-CD20 monoclonal antibody approved by the FDA for the treatment of the relapsing-remitting and active secondary progressive forms of the disease [3]. Although clinical trials of ocrelizumab in rheumatoid arthritis and lupus were halted due to high rates of serious infections, these adverse effects were not seen in trials in people with MS [7,8]. However, there is accumulating evidence from published case series demonstrating colitis and inflammatory bowel changes, which can be presumably associated with Ocrevus given temporal plausibility [4-6]. The average time to onset of colitis from the start of Ocrevus therapy ranges from one week to five years [4]. These articles have discussed patients’ medical histories, including treatments attempted before Ocrevus [4]. However, to date, there have been no cases reported in the literature of the possible compounded effect of surgical intervention on a patient receiving Ocrevus infusions in association with subsequent postoperative severe localized colitis. This case brings to light a new factor to consider in patients receiving treatment with immunomodulators who undergo surgical intervention. It has been proposed that immunomodulators that act to deplete B cells, such as Ocrevus, have the potential to predispose patients to autoimmunity through dysregulation of the immune system, and that this can occur in the background rather asymptomatically until an immunological trigger or disruption catalyzes the development of the autoimmune process.

## Conclusions

Reported cases of colitis in Ocrevus use have postulated possible triggers to be a cumulative effect of the medication from repeat infusions or simply a delayed presentation of the colitis in relation to beginning therapy with the drug. This case proposes surgical intervention as a new possible trigger, heralding a more localized colitis picture in the setting of Ocrevus use. Drawing awareness of this offers physicians and patients with MS a better understanding of possible postoperative complications they may be at higher risk for and allows physicians to be prepared should patients return after a relatively common procedure.

## References

[1] Lehmann-Horn K, Kronsbein HC, Weber MS (2013). Targeting B cells in the treatment of multiple sclerosis: recent advances and remaining challenges. Ther Adv Neurol Disord.

[2] Roos I, Hughes S, McDonnell G (2023). Rituximab vs ocrelizumab in relapsing-remitting multiple sclerosis. JAMA Neurol.

[3] Mulero P, Midaglia L, Montalban X (2018). Ocrelizumab: a new milestone in multiple sclerosis therapy. Ther Adv Neurol Disord.

[4] Sunjaya DB, Taborda C, Obeng R, Dhere T (2020). First case of refractory colitis caused by ocrelizumab. Inflamm Bowel Dis.

[5] Kim T, Brinker A, Croteau D (2023). Immune-mediated colitis associated with ocrelizumab: a new safety risk. Mult Scler.

[6] Lee HH, Sritharan N, Bermingham D, Strey G (2020). Ocrelizumab-induced severe colitis. Case Rep Gastrointest Med.

[7] Mysler EF, Spindler AJ, Guzman R (2013). Efficacy and safety of ocrelizumab in active proliferative lupus nephritis: results from a randomized, double-blind, phase III study. Arthritis Rheum.

[8] Stohl W, Gomez-Reino J, Olech E (2012). Safety and efficacy of ocrelizumab in combination with methotrexate in MTX-naive subjects with rheumatoid arthritis: the phase III FILM trial. Ann Rheum Dis.

